# Anesthetic Considerations for a Patient With Hereditary Hemorrhagic Telangiectasia (Osler–Weber–Rendu Syndrome) Undergoing a Five-Box Thoracoscopic Maze Procedure for Atrial Fibrillation

**DOI:** 10.1177/2324709614553669

**Published:** 2014-10-10

**Authors:** Dominic Robinson, Barbara Rogers, Ritu Kapoor, Joseph Swan, Gaylynn Speas, Rebecca Gutmann

**Affiliations:** 1 The Ohio State University Wexner Medical Center, Columbus, OH, USA

**Keywords:** hereditary hemorrhagic telangiectasia, Osler–Weber–Rendu syndrome, five-box maze procedure, arteriovenous malformation

## Abstract

Hereditary hemorrhagic telangiectasia (HHT) is an autosomal dominant genetic disorder involving the abnormal communication of vascular structures. HHT typically presents with recurrent epistaxis and telangiectasis of the nasal and buccal mucosa, tongue, and lips. More serious manifestations of this disease include cerebral, pulmonary, gastrointestinal, and hepatic arteriovenous malformations. This case report details a 55-year-old male with HHT undergoing a five-box maze procedure for curative treatment of atrial fibrillation. Particular anesthetic considerations are described to reduce morbidity and mortality in this patient population.

## Introduction

Hereditary hemorrhagic telangiectasia (HHT) is an inherited genetic disorder involving the abnormal communication of vascular structures—specifically a malformation between arteries and veins. Incidence of this disease process has been reported from 1:5000 to 1-2:100 000.^1-3^ Telangiectasias refer to malformations of small vessels and can be described as dilated arterioles in communication with dilated post capillary venules.^3^ The structure of these vessels are often convoluted with a single layer of endothelium on a continuous basement membrane.^4^ HHT also can lead to development of large vascular malformations known as arteriovenous malformations (AVMs). AVMs in conjunction with dilated microvessels often lead to issues of bleeding, most commonly epistaxsis. Other manifestations of the disease process include AVMs in the pulmonary, hepatic, and cerebral circulations, leading to complications such as hemorrhage and embolism leading to stroke. Arteriovenous malformations are more prone to hemorrhage due to the fragile structure of the vessels.^2^ Diagnosis of this disease has classically been established by the clinical triad of HHT: epistaxis, telangiectasias, and family history. More recently, the Scientific Advisory Board of the HHT Foundation International Inc has aided to establish The Curacao Criteria, a consensus diagnostic clinical criteria for HHT. The Curacao Criteria stratifies the diagnosis into 3 categories: definite, possible, or unlikely. Criteria for diagnosis include epistaxis, telangiectasias, visceral AVM lesions, and family history (see Table 1).^5^

**Table 1.** Diagnostic criteria for HHT.

Diagnosis of HHT	Criteria Present
Definite	3-4
Possible	2
Unlikely	0-1
Criteria	Description
Epistaxis	Spontaneous and recurrent nose bleeds
Telangiectasias	Multiple at sites such as nasal and buccal mucosa, lips, tongue, pharynx, nose, hands
Visceral AVMs	Examples: pulmonary, hepatic, gastrointestinal, cerebral, or spinal
Family history	First-degree relative with HHT

Abbreviations: HHT: hereditary hemorrhagic telangiectasia; AVM: arteriovenous malformation.

HHT has an autosomal dominant inheritance pattern, as such disease manifestations occur in the heterozygous form. Reports over homozygous inheritance for this disease process have been controversial, rare, and thought to be incompatible with life.^1^ There have been over 100 mutations reported for 3 genes responsible for causing the disease.^1^ There are 5 genetic subtypes of HHT, 3 of which have been linked to specific genes (see Table 2).^2^ The endoglin gene located on chromosome 9q is responsible for HHT-1 subtype. The activin receptor like kinase (ALK-1) gene on chromosome 12q is associated with HHT-2. Both genes encode type I glycoprotein cell surface receptors and are part of the TGF-beta superfamily of ligands. Finally, the HTJP genetic subtype is linked to the SMAD4 gene. The SMAD4 protein is an intracellular signaling modulator for the TGF superfamily receptors.^6-8^ Mutations in this gene cause HHT as well as juvenile polyposis.^7^ Genetic tests are available for the endoglin, ALK-1, and SMAD4 associated gene mutations.^9^

**Table 2.** Genetic characteristics for HHT.

Genetic Subtype	Gene	Protein	Chromosome	Genetic Testing
HHT1	ENG	Endoglin	9q	Yes
HHT2	ACVRL1	ALK-1	12q	Yes
HHT3	Unknown	Unknown	5q	No
HHT4	Unknown	Unknown	7p	No
HTJP	SMAD4	SMAD4	18q	Yes

HHT typically presents with recurrent epistaxis and telangiectasis of the nasal and buccal mucosa, tongue, and lips.^3,9,10^ As previously mentioned, more serious manifestations of this disease include cerebral, pulmonary, gastrointestinal, and hepatic AVMs.

Epistaxis is the most common manifestation of the disease, occurring in 93% of patients with HHT. Various techniques have been described for the treatment of epistaxis in the setting of HHT including embolization of the nasal vasculature, laser photocoagulation, septodermoplasty, brachytherapy, surgical repair with autograft, hormonal therapy, as well as simple measures such as cautery and packing.^3,11^

Pulmonary vascular complications associated with HHT can be significant and life threatening. The most common pulmonary manifestation are AVMs, which are present in 15% to 40% of cases.^9,12^ Weingarten et al identified over 50% of patients having pulmonary AVMs in a retrospective study from a cohort of 74 patients with HHT undergoing general anesthesia at the Mayo Clinic in Rochester, Minnesota.^10^ Significant sequella of pulmonary AVMs include hemoptysis, hemothorax, and thromboembolic disease (transient ischemic attack or stroke) from right to left shunting through the AVM. Also, pulmonary hypertension may commonly manifest secondary to high cardiac output failure due to AVMs in the liver, typically with patients with large hepatic arteriovenous fistulas (AVF). Shunting of blood through the AVF results in a decrease in atrial pressure leading to a reduction in left ventricular afterload, systemic venous resistance, and venous return. This leads to a compensatory increase in cardiac output by increases in stroke volume and heart rate.^13^

## Case Report

A 55-year-old male with history of atrial fibrillation, HHT, hyperlipidemia, hypertension, prostate cancer status post prostatectomy, remote smoking, and obesity was scheduled for a Maze procedure for treatment of atrial fibrillation. Medications for this patient included estradiol TD nasal spray, cromolyn sodium nasal spray, mupirocin 2% nasal ointment, aspirin, digoxin, metoprolol, and pravastatin.

Preoperatively he was seen by Hematology for assessment of the use of anticoagulation therapy for atrial fibrillation in the setting of HHT. As mentioned, this patient was currently on only aspirin for anticoagulation given the risk of bleeding with HHT. After consultation, recommendations were to avoid further anticoagulation unless proved to be medically necessary in the setting of his cardiac pathology. Further recommendations revealed close follow-up with his physician as well as ongoing laboratory markers to evaluate for anemia.

The patient was also seen preoperatively in the Outpatient Preoperative Assessment Center in preparation for his surgery. Pertinent physical exam findings at that time revealed several telangiectasias on the tongue, lips, and right cheek. Exam of the airway revealed a mallampati class II, full cervical range of motion in all planes, and aforementioned small focal telangiectasias of the tongue but none noted in the posterior oropharynx. The patient was determined to be an ASA physical status 3 with good cardiac functional capacity. He also was determined to be high risk for obstructive sleep apnea as determined by the STOP BANG questionnaire.^14^

Laboratory data included a hemoglobin of 15.1 g/dL, hematocrit of 43.6%, and electrocardiogram showing atrial fibrillation with a rate of 68. A chest x-ray reviled no cardiopulmonary pathology. There was a recent transthoracic echocardiogram completed revealing an ejection fraction of 59% with left ventricular hypertrophy and a mildly dilated left atrium. There were no signs of diastolic dysfunction, and the heart was otherwise structurally normal. Furthermore, there was also documented evidence of a negative lexiscan cardiolyte stress test preformed 3 months prior.

The patient was originally diagnosed with atrial fibrillation 8 years ago and had undergone successful cardioversion to normal sinus rhythm after his initial presentation. He had spontaneously converted back to atrial fibrillation approximately 5 months prior to his planned surgery and had since been experiencing ongoing dyspnea on exertion and palpitations.

Significant to his history of HHT, he previously had experienced frequent episodes of epistaxis and does follow with an otolaryngologist. His epistaxis was complicated by a deviated septum in the setting of HHT, and he subsequently underwent septoplasty for surgical repair. After this nasal surgery he was started on a nasal estrogen for treatment of his epistaxis. Estrogens have been shown to be beneficial in the treatment of epistaxis secondary to HHT. It is postulated that estrogens may improve the integrity of the vascular endothelium or cause microhemostasis in the microcirculation.^15^ Since initiation of treatment and nasal surgery the patient’s episodes of epistaxis have been rare.

On arrival in the preoperative holding area, an 18 gauge IV was started and a right radial arterial line was inserted for hemodynamic monitoring. The patient was transferred to the operating room where standard ASA monitors were applied and pre-oxygenation commenced. General anesthesia was induced with fentanyl, midazolam, lidocaine, propofol, and rocuronium. The patient was intubated with a left double lumen endotracheal tube (ETT) using a MAC 3 blade revealing a Cormack-Lehane grade I view. Careful attention with gentle manipulation was used during direct laryngoscopy and no telangiectasias were appreciated in the posterior oropharynx. The ETT was pretreated by soaking in warm sterile normal saline to allow pliability and lubricated to facilitate smooth intubation and avoid airway trauma and rupture of any possible oropharyngeal telangiectasias. After successful intubation, correct positioning of the ETT was confirmed by bronchoscopy. After induction of general anesthesia, a cordis centeral venous catheter was inserted in the right internal jugular vein for central venous pressure monitoring and access for fluid and blood resuscitation if needed. A transesophageal echocardiogram (TEE) was performed ruling out an intracardiac thrombus or any significant structural heart disease. General anesthesia was maintained with isoflurane, oxygen, fentanyl, and vecuronium. Single lung ventilation ensued on the left for access to the heart via thoracotomy on the right chest wall. Prior to induction, the patient was in atrial fibrillation with a rate of 111. Intraoperatively successful cardioversion was performed 3 times for rates in the 160s. The surgery was successful without complication, and the patient remained in normal sinus rhythm with a rate of 70 at the conclusion of the procedure. Near the end of the procedure 2 lung ventilation resumed and the patient was treated with ondansetron for prevention of postoperative nausea and vomiting. The patient’s neuromuscular blockade was reversed with neostigmine and glycopyrolate. The double lumen ETT was exchanged for an 8.0 single lumen ETT under direct visualization with a size 4 McGrath video laryngoscope. Due to the high risk for obstructive sleep apnea (OSA) the decision was made to transfer the patient to the intensive care unit intubated where he was able to recover from anesthesia and was successfully extubated later in the day. Estimated blood loss from the case was 50 cc, urine output was determined to be 200 cc, and the patient received a total of 2700 cc of crystalloid throughout the procedure.

## Discussion

Patients with HHT may present for procedures related to their pathology such as angiography and embolizations secondary to AVMs or for unrelated surgeries dealing with comorbid disease.^4,16-18^ To the best knowledge of the authors, this is the first reported case of a patient with HHT undergoing a five-box thorascoscopic maze procedure. There are specific anesthetic considerations associated with patients that have HHT and undergoing this procedure.

The gold standard for surgical treatment of atrial fibrillation has long been known as the Cox-Maze III procedure, first performed over 20 years ago. The basic premise of the maze procedure is to eliminate atrial fibrillation by using incisional scars to block abnormal electrical circuits of the heart. This procedure does have its limitations as it was originally performed with an arrested heart requiring cardiopulmonary bypass and an open sternotomy approach. With advances in surgical techniques, in 2003 Randall Wolf developed the “Wolf Mini-Maze” procedure that allowed for a minimally invasive approach without the need for cardiopulmonary bypass as well as intraoperative verification of electrical isolation.^19^ Limitations with this procedure have been reported as incomplete curative treatment for all etiologies of atrial fibrillation as it does not address the left atrium, which can be a source for conduction abnormalities associated with this disease process.^20^ The five-box maze procedure, described by Sirak et al, is a more recent application with reported curative rates for atrial fibrillation in 94% of cases.^21^ This procedure has become a novel technique that does address the entire anatomy of the heart and all mechanisms responsible for atrial fibrillation in a minimally invasive way, without need for cardiopulmonary bypass, and allows for verification of electrical isolation of all potential sources intraoperatively.^20^

It is well known that atrial fibrillation increases the risk of thromboembolic stroke secondary to clot formation in the atria due to lack of appropriate atrial contraction and flow. Consequently, patients with atrial fibrillation often require treatment with anticoagulation for preventative measures. This patient had a CHADS2 score of 1 and was treated with aspirin. HHT can be described as a relative contraindication to warfarin therapy because of the obvious risk of bleeding. In patients with CHADS2 scores of 2 or higher, it would be wise to use a multidisciplinary approach with expert consultation to weight the risks versus benefits of anticoagulation in the patient with HHT.

As the most common occurrence of telangiectasias are in the oral cavity and nasopharyngeal passages, bleeding from the oropharynx is an initial concern in the anesthetic management of patients with HHT. Rupture of a known or unknown AVM during direct laryngoscopy and passage of the endotracheal tube may lead to bleeding causing obstruction of a view of the vocal cords potentially causing a difficult airway situation. Measures to optimize conditions include gentle direct laryngoscopy, lubrication of the ETT, using a smaller size ETT if appropriate, and soft gentle suction to prevent trauma. A double lumen tube was required for selective one lung ventilation with this procedure. This did present a potential challenge as a larger sized tube can pose a greater risk of trauma and rupture of AVMs in the patient with HHT.

As described in this case, induction and maintenance of anesthesia were performed with anesthetics known to be cardioprotective and provide stability to myocardial function. The induction dose of propofol was reduced and lidocaine and fentanyl were used to blunt the sympathetic response of direct laryngoscopy, preventing abrupt increases in blood pressure that could potentially lead to rupture of vascular malformations. Isoflurane was chosen as the inhalation agent for its ability to vasodilate the coronary arteries allowing adequate flow to the myocardium.

Thrombus formation leading to embolic disease as well as shunting of air through potential large AVMs in the pulmonary or gastrointestinal circulation also pose anesthetic concern and consideration. Vigilance in removing all air from syringes and intravenous tubing as well as placing air filters near the hub of the tubing can help prevent introducing air into the venous circulation preventing embolic pathology.

Consideration for invasive monitoring in the esophagus should be taken into account for patients with AVMs of the gastrointestinal tract. Risks and benefits for use of invasive monitoring and interventions such as nasogastric or orogastric tubes, esophageal Doppler, esophageal stethoscopes, and TEE monitoring should be taken into careful consideration prior to use. Rupture of an AVM of the gastrointestinal tract could lead to significant morbidity.

Furthermore, a thorough and adequate preoperative evaluation including assessment of hemoglobin, hematocrit, platelet count, and coagulation profile should be undertaken. Special attention to concurrent anticoagulation medications and other comorbid disease states causing a propensity to bleed should be screened. The surgical patient with HHT should be medically optimized prior to undergoing invasive intervention. If clinically warranted, screening surgical patients for visceral AVMs in the brain, lungs, liver, and gastrointestinal tract should be considered.^18^ In patients demonstrating a diagnosis of HHT in the setting of comorbid disease requiring anticoagulation therapy, as in this case, expert consultation should be requested. Careful consideration to each of these measures can go a long way in reducing morbidity and mortality in this patient population.
